# Insights into role of microRNAs in cardiac development, cardiac diseases, and developing novel therapies

**DOI:** 10.22038/ijbms.2020.40974.10015

**Published:** 2020-08

**Authors:** Maedeh Arabian, Fatemeh Mirzadeh Azad, Majid Maleki, Mahshid Malakootian

**Affiliations:** 1 Cardiogenetic Research Center, Rajaie Cardiovascular, Medical, and Research Center, Iran University of Medical Sciences, Tehran, Iran; 2 Department of Molecular Genetics, Faculty of Biological Sciences, Tarbiat Modares University, Tehran, Iran

**Keywords:** Cardiac development, Cardiovascular diseases, MicroRNAs, Non-coding RNAs, RNA-based therapy

## Abstract

MicroRNAs (miRNAs) are a subfamily of small noncoding RNAs that play a variety of roles in regulating gene expression in nearly all organisms. They affect different biological pathways by post-transcriptionally regulating mRNAs. Aside from miRNAs’ role in maintaining cellular homeostasis, their perturbation is related to several pathologic states and diseases. Cardiovascular disorders are considered some of the most mortal multifactorial diseases that are caused by the deregulation of network of genes and effects of environmental factors. In this review, we discuss the role of miRNAs in cardiac homeostasis and malfunctions. We reviewed published research on association and role of miRNAs in cardiac development and diseases and investigated the possible links between regulatory miRNAs and different cardiac disorders. Research shows that manipulating miRNAs expression affects the integrity and functionality of the cardiovascular system. Moreover, deregulation of miRNAs, is observed in many cardiac diseases. These findings intensify the pivotal role of miRNAs in the development and speciﬁc pathological disorders of the cardiovascular system. In this review, we summarized the latest findings on the involvement of miRNAs in cardiac development, and continued by their role in congenital heart diseases and rheumatic heart disease, which are some of the leading causes of infant death and cardiovascular morbidity and mortality. Considering the significance of miRNAs in cardiac homeostasis and malfunctions, they are considered as promising therapeutic targets in cardiovascular diseases.

## Background

Each year, more than 32,000 newborns are born with various forms of heart malformations (1 out of every 125 to 150). Cardiovascular diseases constitute the principal cause of morbidity and mortality in adults (1). A double pump comprising 4 chambers, the heart, is one of the earliest differentiating and functioning organs and the most vulnerable organs to birth defects (2, 3). 

The proper development of the heart is dependent on a sophisticated network of crosstalk between multiple cell lineages; cardiomyocytes, endothelial cells, fibroblasts, epicardial cells, neural crest cells, and smooth muscle cells. This coordination occurs via paracrine interactions, cell–ECM interactions, and cell–cell interactions in order to facilitate survival, growth, proliferation, differentiation, and migration of cardiac tissue(4, 5). Consequently a myriad of genes that encode various transcription and growth factors, adhesion molecules, structural proteins, signal molecules, and ion channels regulate cardiac morphogenesis. 

Briefly, the key points of cardiac development start from undifferentiated mesoderm with interfering of bone morphogenetic proteins (*BMP*), wingless-related integration site family member (*WNT*), and fibroblast growth factors (*FGF*) changing to cardiac mesoderm. Following this NK2 Homeobox 5 (*NKX2.5*), GATA-binding factor (*GATA*), Myocyte enhancer factor-2 (*MEF2*), T-box transcription factor (*TBX*), Heart- and neural crest derivatives-expressed protein (*HAND*), pituitary homeobox-2 (*PITX2*), MEF2C, Spalt Like Transcription Factor-4 (*SALL4*), Serum response factor (*SRF*), zinc finger protein, FOG family member 2 (*FOG2*), Cbp/p300 interacting transactivator with Glu/Asp rich carboxy-terminal domain 2 (*CITED2*), and Zic family member 3 (*ZIC3*) transcription factors participated in development of cardiac tube, looping, cardiac chambers, and formation of cardiac valves, great vessels, and their relationships (6-8). Therefore, numerous control mechanisms regulate and integrate the diverse functions and component parts of the cardiovascular system. Misregulation of these processes leads to developmental diseases and pathological phenotypes.

The collective work of several research groups has highlighted that the non-protein coding parts of the genome, formerly believed to be junk DNA, might also be of functional importance. Research in the last decade has established that microRNAs (miRNAs), an evolutionary highly conserved class of non-coding RNAs (ncRNAs), compose the main part of the human small noncoding RNAs in transcriptome. In addition, given their highly organ- and cell-particular expression patterns, miRNAs are postulated to participate in regulating protein-coding gene expression in development and disease. Indeed, many research groups have recently unraveled the astonishingly powerful role of miRNAs in the development/diseases of the cardiovascular system (9-12). Here, we review the role of miRNAs in cardiac development as well as congenital and rheumatic heart disease (RHD) (Table 1). In developing countries, RHD stands as one of the prominent causes of cardiovascular morbidity and mortality. We reviewed the latest findings on the role of miRNAs in RHD. Then, we also considered the therapeutic roles of miRNAs in order to develop the novel miRNA therapeutics for management of the cardiovascular diseases.

***Biogenesis of miRNAs***

Since the year 1993, when 2 research groups introduced the first non-coding miRNA-namely, lin-4-and its interaction with the complementary sequence existing in the untranslated region of lin-14 messenger RNA (mRNA) in the nematode *Caenorhabditis elegans* (13, 14) , many investigators have identified copious miRNAs in the human genome. Up to now, 28 645 miRNAs have been submitted to the miRBase database (http://www.mirbase.org/). These tiny ncRNAs fulfill essential roles in regulating a broad range of physiological and pathological circumstances. Manufactured from hairpin-shaped transcripts, miRNAs are endogenous single-stranded ncRNAs that are 18 to 24 nucleotides in length. miRNAs modulate transcriptional and post-transcriptional gene expression via interaction with complementary target sites in targeted genes. It has been estimated that the expression of more than 60% of protein-coding genes is modulated by miRNAs (15). Consequently, in response to pathophysiological phenomena, these small ncRNAs act as the fine-tuners or micromanagers of gene expression.

**Figure 1:**
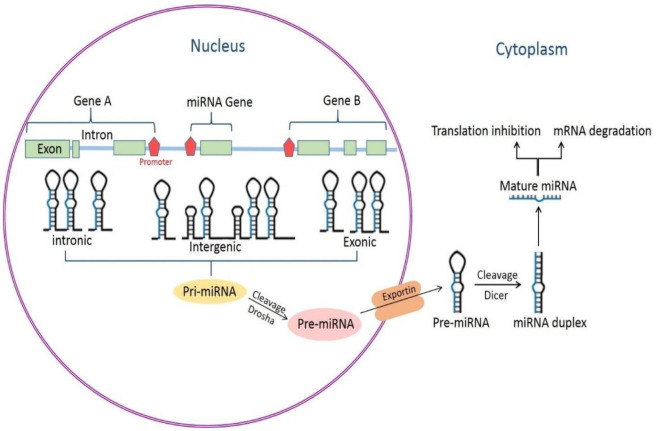
Biogenesis of microRNAs (miRNAs)

The genes of miRNAs, whether a single gene or a cluster of genes, can be positioned in the intergenic, intronic, or exonic regions of protein-coding or noncoding genes in the genome [8] and are mainly transcribed by RNA polymerase II (16). They may be under the control of their own miRNA-specific promoters or under the direction of the promoters of their host genes (17)(Figure 1). The primary transcript could be several thousand nucleotides long and contain several miRNA precursors. miRNAs that reside in 1 primary transcript are known as a cluster. miRNA precursors form a stable stemloop structure in primary transcript RNAs, which are recognized by the microprocessor complex, Pasha (RNA-binding protein DGCR8), and the ribonuclease III enzyme, Drosha(18). After being cut by Drosha, an miRNA-producing stemloop (now known as a pre-miRNA) is joined by exportin 5 and leaves the cell nucleus in a Ran-guanosine-5’-triphosphate-dependent manner. The RNase III enzyme Dicer is in charge of the cytoplasmic processing of pre-miRNAs and produces 18- to 22-nucleotide long mature miRNAs. Mature miRNAs then couple with the RNA-induced silencing (RISC) complex and execute their effect by base-pairing with the target mRNA (19, 20). The extent of the base-pairing miRNA to the target determines the fate of the target. In this context, if the miRNA has perfect or near-perfect complementarity to the targeted mRNA, it directly destroys the mRNA (21). Alternatively, an imperfect base-pairing between miRNAs and target mRNAs actuates the suppression of protein translation without strongly affecting mRNA levels (11) (Figure 1).

***miRNAs and the development of the heart***

Tissue-specific omission of the Dicer gene, which encodes an RNase III endoribonuclease that is necessary for miRNA processing, has revealed that miRNAs are indispensable for cardiovascular development in mice. After Dicer deletion, lethal phenotypes are observed in myocardial and vascular lineage (10, 22, 23). Additionally, the deletion of Dicer in the myocardium is allied to a high incidence of death, cardiac hypertrophy, and reactivation of the cardiac gene program (24, 25). Therefore, a mature heart requires Dicer activity in order to have a normal function.

Recently, accumulative studies have ascertained the participation of these small RNAs in multiple cardiovascular processes such as embryonic stem-cell differentiation, cardiomyocyte identity, contractility, ion-channel regulation, and cardiac conduction. Molecular regulation of cardiomyocyte differentiation contributes to a major period in early heart development, as well as adult tissue homeostasis, and the network of miRNAs that govern these biological steps have been well reviewed in it (26, 27). Accordingly, precise and complex connections among various cells from distinct lineages: cardiomyocytes; endocardial, epicardial, and vascular cells; fibroblasts; and cells of the conduction system are required to form a healthy heart (12). miRNAs have been recognized in the cardiac tissue at all points of development; moreover, not only are they enriched in different cardiac cell types but also they participate in the specification of cell identity (28-31).

miRNAs that are specially expressed or spotted in cardiac, smooth, and skeletal muscles are termed “myo-miRNAs” or “muscle miRNAs” (32). Among them, 4 specific miRNAs-namely miR-1, miR-133, miR-208, and miR-499-are extremely expressed in the different stages of heart development. Several non-myo-miRNAs have also been implicated in the development and differentiation of the heart.

***miR-1 and miR-133 families***

miR-1, the first miRNA involved in cardiac development, is the prominent miRNA in cardiac myocytes and skeletal muscles. It is highly conserved from fruit flies to humans. The gene-encoding miR-1 and miR-133 family members are clustered and co-expressed from a common bicistronic unit. Thus far, research has revealed 3 clusters that are spawned in 3 different chromosomal regions in the human genome and fabricate miR1-1, miR-133a-1, miR-1-2, miR-133a-2, miR-133b, and miR-206. The miR-1-1/miR-133a-2 and miR-1-2/miR-133a-1 clusters are intronic and they are positioned at 20q13.33 and 18q11.2, respectively. miR-206/133b is in an intergenic region and placed at 6p12.2. This cluster is homologous to miR-1, but is expressed exclusively in skeletal muscles (33). Although miR-133-a1 and miR-133-a2 have the same mature sequences, a single nucleotide at the 3’ end of miR-133b differs from the 3’ end of miR-133a (28). miR-133a is enriched in striated muscles (34). The expression of miR-1 and miR-133 in the heart and skeletal muscles is under the control of the serum response factor/myocardin and the myocyte enhancer factor-2/myogenic transcription factors, respectively (35). Furthermore, the balance between the differentiation and proliferation of cardiac precursor cells is controlled by miR-1, which targets the transcription factor Hand2, which is involved in promoting the augmentation of ventricular cardiomyocytes (35). In addition miR-1 targets the histone deacetylase (HDAC) 4, handtranscription factor (Hand)2, connexin 43 (GJA1), and K^+^ channel subunit Kir2.1 (KCNJ2) transcripts which are important for cardiac development or function (36). miR-1 and miR-133 act cooperatively to suppress the differentiation of embryonic stem cells into the endoderm and ectoderm and promote mesoderm differentiation. In the following cardiac lineage, miR-1 continues the promotion of cardiomyocyte differentiation, whereas miR-133 impedes the process (37). 

***miR-208 and miR-499 families***

miR-208a, miR-208b, and miR-499 are intronic miRNAs that are one to one placed in the intron of 3 muscle-specific myosin genes-namely *Myh-6*, *Myh-7*, and *Myh-7b*. 

The main determinant of the efficiency of muscle contraction is dependent on the myosin heavy chain (MHC), the major contractile protein of cardiac and skeletal muscle cells. The expression of 2 MHC genes-namely *α-MHC* and *β-MHC*-which are connected in a head-to-tail orientation and regulated in an antithetical mode, provides cardiac contractility (38). 

Mounting evidence from *in vitro* modeling experiments has revealed that the expression of these miRNAs is concomitant with the expression of their corresponding host genes during development (32, 39, 40). A collection of transcriptional repressors and signaling molecules governs the MHC expression. Similarly, the activity of the thyroid hormone and the stress-responsiveness of cardiac muscle cells are regulated by these myo-miRNAs (39-41). While miR-208b is chiefly expressed in atrial cells, miR-208a is mostly expressed in ventricular cells of the adult human heart.

The family members of miR-208, miR-208a, and miR-208b share identical seed sequences. miR-499, which shares 6 overlapping nucleotides in the seed region, is extremely similar to miR-208a and miR-208b*.* These close similarities in seed sequences imply that they probably regulate a similar array of target genes (42). 

***Cardiac-related miRNAs***

Recent research has shed light on the vital role of the miR-17-92 cluster during *in vitro* differentiation of cardiac progenitor cells into cardiomyocytes and the proliferation of cardiomyocytes in embryonic and adult hearts (43, 44). This cluster is placed in the long arm of human chromosome 13, and the pri-miR-17-92 transcript is processed into distinct mature miRNAs comprising miR-17-5p, miR-17-3p, miR-18a, miR-19a, miR-19b, miR-20a, and miR-92a. Additionally, the miR-17-92 cluster regulates bone morphogenetic protein (BMP) signaling, which is involved in myocardial differentiation from cardiac progenitors (43). 

The overexpression of one of the members of this family, miR-20a, suppresses differentiation and proliferation and simultaneously enhances apoptosis via regulating the Hedgehog (Hh) signaling pathway, which prompts cardiac differentiation in cardiac embryonic development (45). The ectopic expression of miR-17-92 represses Friend of GATA-2 expression in embryonic cardiomyocytes (46). Friend of GATA-2, also termed “ZFPM2”, is a cofactor for GATA transcription factors and is required for heart development (47).

The miR-15 family, which has 6 highly conserved miRNAs—namely miR-195, miR-15a, miR-15b, miR-16-1, miR-16-2, and miR-497—spawned on 3 different chromosomes, is involved in cardiomyocyte proliferation and induces embryonic cardiomyocyte mitotic arrest through targeting multiple cell-cycle regulators (48).

miR-21 is expressed in the cardiac valve endothelium during development and is implicated in cardiac valvulogenesis via down-regulating the tumor suppressor Pdcd4b in the course of valve development (49). In addition, miR-196a, which is spotted at gestational age 12 to 14 weeks, controls homeobox B8/sonic hedgehog signaling and is implicated in cardiac septation, morphogenesis, and valve formation (50).

***miRNAs in congenital heart diseases***

Structural problems of the heart existing at birth are known as congenital heart anomalies or congenital heart diseases. Ventricular septal defects, tetralogy of Fallot, atrial septal defects, patent ductus arteriosus, pulmonary valve atresia, coarctation of the aorta, and tricuspid atresia are among the numerous types of congenital heart diseases (51). Congenital heart diseases constitute the principal cause of morbidity and mortality in infants with heart problems (52, 53). Efforts toward understanding the cellular and molecular mechanisms of cardiac anomalies can reduce the prevalence of congenital heart defects.

miR-1-1 and miR-181c are implicated in the pathogenesis of ventricular septal defects (54). The expression level of miR-1-1 is decreased in patients with ventricular septal defects and is associated with a rise in the expression level of the gap junction protein alpha 1 (GJA1) and SOX9 proteins. Moreover, a down-regulation in the level of the bone morphogenetic protein receptor type II (BMPR2) was reported to be correlated with an elevated expression of miR-181c in affected tissues compared to healthy control tissues (54-56). In addition, the ectopic expression of miR-1 in the developing heart reduces the pool of proliferating ventricular cardiomyocytes via regulating the Hand2 transcription factor, which promotes ventricular cardiomyocyte expansion (35). 

In one study, the circulating miRNA profile of patients with ventricular septal defects revealed that 7 miRNAs-namely let-7e-5p, miR-155-5p, miR-222-3p, miR-433, miR-379-5p, miR-409-3p, and miR-487b-were down-regulated and miR-498 was up-regulated in comparison to the normal control group (57). These miRNAs could be underlying the congenital abnormalities and VSD since they target NOTCH1, HAND1, GATA3 and ZFM2 factors (58). 

In 2012, O’Brien *et al*. reported 61 miRNAs with significant alterations in expression levels in the myocardium of children with tetralogy of Fallot, in comparison with normally developing comparison subjects (59). The perturbation of miR-421 expression has a negative impact on SOX4, a key regulator of the Notch and Wnt signaling pathways, which is implicated in cardiac development and contributes to congenital defects. In an investigation, excessive expressions of miR-421 were observed in the right ventricles of infants with tetralogy of Fallot (60). The dysregulation of miR-196a could play a role in the formation of atrioventricular septal defects and cardiac valve dysfunction via regulating the HOXB8-Shh signaling (50). Although the expression of miR-133a-1/miR-1-2 and miR-133a-2/miR-1-1 genes is detectable throughout the ventricular myocardium and interventricular septum from E8.5 until adulthood, the deletion of miR-133a-1 or miR-133a-2 does not have obvious effects on cardiac development. However, the absence of both miRNAs leads to late embryonic and neonatal lethality owing to ventricular septal defects and chamber dilatation (61, 62). Likewise, the targeted omission of the miR-17~92 cluster causes similar cardiac abnormalities (63). 

Some patients with Down syndrome and DiGeorge syndrome have congenital heart diseases as well. The overexpression of 5 miRNAs-namely miR-99a, let-7c, miR-125b-2, miR-155, and miR-802-which are located on human chromosome 21, has been found in the cardiac tissues of patients with Down syndrome (36, 64, 65). Interestingly, the critical region on chromosome 22 (22q11.2), which encodes a component of the RISC complex and is crucial for miR biogenesis is deleted in DiGeorge syndrome, which is due to the haploinsufficiency of this disease. The dysregulation of miR expression may, therefore, play a role in the gene dosage sensitivity of this disease via impacting many miR targets (66, 67).

***miRNAs in rheumatic heart disease***

Rheumatic heart disease is an autoimmune response to acute rheumatic fever (68), which occurs as a result of a bacterial infection with streptococcal bacteria (69). The heart could be inflamed with recurrent and repeated episodes of acute rheumatic fever, triggering the chronic inflammation of the myocardium and endocardium, hemodynamic alteration, valvular dysfunction (70) , and finally heart failure, stroke, arrhythmias, endocarditis, or other related complications. Patients with rheumatic heart disease are commonly diagnosed too late with severe and irreversible valvular dysfunction, which may necessitate cardiac surgery. Therefore, a specific diagnostic method is required to start effective therapies for these patients at the early stage of the disease. Finding novel biomarkers can confer early detection and characterization of the pathophysiology of rheumatic heart disease with a view to avoiding cardiac surgery (71). 

Recently, as is the case with other cardiovascular diseases, research has highlighted the role of miRNAs in the diagnosis and treatment of rheumatic heart disease. In this regard, the results obtained through miRNA array have shown that miR-1183 and miR-1299 are significantly up-regulated in these patients. Moreover, increased levels of miR-1183 and miR-1299 expression have been found either in the plasma or in the tissues of patients with rheumatic heart disease, which confirms that these miRNAs can be considered potential biomarkers in this autoimmune response (72). It should be noted that the expression of miR-1183 in patients suffering from rheumatic heart disease with a high pulmonary artery systolic pressure is different from that in patients with a low pressure. This difference could suggest that the overexpression of miR-1183 is due to the remodeling of the pulmonary artery secondary to rheumatic heart disease. Such findings show that the role of miR-1183 in secondary disease is more prominent than that in primary complications. An investigation discovered that miR-1299 could regulate rheumatic heart disease directly (72). A novel investigation on miRNAs participated in atrial fibrillation due to rheumatic heart disease revealed the important role of miR-29b-1-5p and miR-29b-2-5p, which act through the down-regulation of various circular RNAs such as circRNA_10998, circRNA_11017, circRNA_11040, and circRNA_11044 (73).

The expression of some miRNAs such as miR-4423-3p, miR-218-1-3p, and miR-101 is significantly down-regulated in patients with rheumatic heart disease (74), and in children with rheumatic carditis miR-16-5p, miR-223-3p, and miR-92a-3p are suppressed (75). miR-101 has a role in regulating the immune system by altering the toll-like receptor 2 (TLR2) expression and through this pathway can suppress the immune system in patients with rheumatic heart disease (74). However, Zhu *et al*. showed that miR-101 functioned as an activator of immune response through reducing mitogen-activated protein kinase phosphatase 1 (MKP1), which modulates the *TLR4* pathway negatively (76). This controversy may be related to the complexity of miRNA action and the specificity of the tissue.

Recently published study using next generation sequencing of the miRNA profile of patients with rheumatic heart disease showed that hsa-miR-205-3p and hsa-miR-3909, are involved in the inflammatory pathway by targeting the IL-1β-IL-1 receptors. These target genes have a crucial role in progression of rheumatic heart disease (77).

Further mechanistic studies should, therefore, be performed to shed light on the modulatory role of miRNAs in the pathogenesis of rheumatic heart disease and to find new diagnostic and treatment approaches (78). 

Some miRNAs that have already been investigated in the other cardiovascular diseases like miR574-5p, miR-208a, miR-208b, and miR-206 (79) could have regulatory roles in rheumatic heart disease, but it is necessary to perform more investigations to confirm their functions.

***miRNA-based therapeutic approaches in cardiovascular disorders***

**Figure 2:**
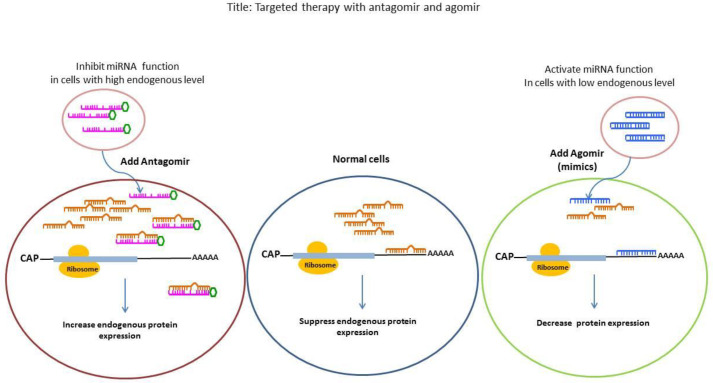
Targeted therapy with antagomir and agomir

In recent years increasing research in the cardiovascular field has revealed the possible role of miRNAs as biomarker and therapeutic targets for the diagnosis and treatment of cardiovascular disorders. In this context, reinstating the miRNA expression levels is the main approach in miRNA-based therapy. Generally 2 different methods have been recognized to modify miRNA expression levels in diseases (Figure 2). Since miRNA activity has either harmful or beneficial effects, the overexpressed miRNAs could be suppressed by antisense oligonucleotides and down-regulated ones could be overexpressed and activated by oligonucleotides mimicking its function for the treatment of diseases. One of the approaches to the up-regulation of miRNAs for therapeutic purposes is the use of adeno-associated viruses (AAVs). The injection of beneficial miRNAs with AAVs into the host leads to a long-lasting expression of the miRNAs *in vivo* (80). In this method, it is necessary to find novel variants of AAVs to target specific cells or tissues. For example, 9, 8, and 6 subtypes of AAVs have cardiotropic effects when applied systemically (81). Another approach is the use of modified synthetic oligonucleotides, which are able to mimic the functions of endogenous miRNAs. The challenging aspects of this method are the design and delivery of double-stranded oligonucleotides. In the inhibitory approach, anti-miRNAs or antagomiRs are applied for the down-regulation of the harmful miRNA. The antagomiRs are single-stranded oligomers that are designed for the targeted miRNA (82, 83). 

From 1983 to 2017, different major biopharmaceutical companies were involved in the development of the therapeutic miRNA molecules. Among them Santaris Pharma from Denmark developed an inhibitor, a short locked nucleic acid (LNA), of miR-122 named miravirsen, which is currently moving toward the market. This miRNA therapeutic, which is the world’s first miRNA-based drug is presently in phase II clinical trials for the treatment of hepatitis C virus (HCV) infection (84, 85).

In addition, several biopharmaceutical companies are developing other miRNA therapeutics for treatment of different diseases, as well as cardiovascular disorders. But all of them are in the preclinical stage and aiming to enter clinical trials (86).

Miragen therapeutics company is working on at least six miRNAs therapeutics related to cardiovascular disorders. In line with this, MGN- 1374, which targets miR-15 and miR-195, is in the preclinical stage for the treatment of post-myocardial infarction. All MGN-2677, MGN-4220, MGN-5804, MGN-6114, and MGN-9103 miRNA-based drugs are in the pipeline of clinical trials. Abovementioned therapeutic drugs target miR-143/145, miR-29, miR-378, miR-92, and miR-208, respectively (40, 87-89). Overall, miRNA-based therapy for cardiovascular diseases could be challenging because the substances that target miRNAs have unique features in terms of nonselective uptake and susceptibility to degradation. Consequently, in this therapeutic approach, it is necessary to design highly stable reagents so as to prevent their degradation during their passage from cellular membranes. 

**Table 1:** Regulatory role of some miRNAs in cardiovascular functions and diseases

**Biological condition and clinical effects**	**miRNAs**	**Function**	**Reference**
Development of heart	miR-1	Promotion of cardiomyocyte differentiation	[31,32]
	miR133 family	Inhibition of cardiomyocyte differentiation	[33,34]
	miR-17-92	Differentiation of cardiac progenitor cell Cardiomyocytes proliferation in embryonic and adult hearts Regulation of Bmp signaling	[40, 41, 42,43]
	miR-208 miR-499	Proliferation of cardiac muscle cells Control the myosin heavy chain	[45]
	miR-20a	Suppression of differentiation and proliferation Increase apoptosis	[40, 42]
	miR-15 family	Cardiomyocyte proliferation Embryonic cardiomyocyte mitotic arrest	[45]
Rheumatic heart disease	miR-1183 miR-1299	Up-regulation in RHD	[65]
	miR-4423-3p miR-218-1-3p miR-101	Down-regulation in RHD	[66, 67]
Congenital heart diseases	miR-1-1	Decreased in VSD patients	[50]
	miR-155-5p miR-222-3p miR-433 miR-487b	Down-regulation in RHD	[51]
	miR-181c miR-498	Elevated expression in RHD	[50,51]
	miR-1	Proliferation of ventricular cardiomyocytes	[33]
	miR-421	Up-regulation in in right ventricle of infants with tetralogy of Fallot	[53]
	miR-196a	Atrioventricular septal defects and cardiac valve dysfunction	[47]
	miR-133a-1/ miR-1-2 miR-133a-2/miR-1-1	Involved in formation of ventricular myocardium and interventricular septum	[54, 55]

RNA polymerase II produces primary transcript RNA transcripts from miRNA genes located in the exonic, intronic, or intergenic regions of noncoding or protein-coding genes. Within the nucleus, Drosha, in association with Pasha (RNA-binding protein DGCR8), cleaves the primary transcript RNAs into precursor miRNAs (pre-miRNAs). A hairpin structure of miRNA, pre-miRNA-with the length of 60 to 100 nucleotides-is actively exported to the cytoplasm via exportin 5. Pre-miRNAs are cleaved by Dicer, creating double-stranded miRNA duplexes in the cytoplasm. Subsequently, these 22 nucleotide duplexes unwind and the guide mature miRNA enters the RNA-induced silencing (RISC) complex accompanied by Argonaute proteins. The binding of the miRNAs to the mRNAs target leads to the inhibition of the translation and/or mRNA degradation

Antagomirs are engineered single-stranded oligonucleotides that inhibit molecules binding to a desired region on the mRNA molecule and specifically are applied to silence endogenous microRNA. Targeting miRNAs by antagomirs in the cells with overexpression of miRNA inhibits and suppresses the function of miRNA and increase expression of target proteins (left side). Agomirs (mimics) are chemically modified double-stranded miRNA that can regulate the function of target gene through the mimicking endogenous miRNA. In the cells with low endogenous level of miRNA, Agomirs lead to derepression of the target proteins (right side). Agomirs and antagomirs have a high affinity for cell membrane and can be richened in target cells and induce specific and stable interfering effects

## Conclusion

In the modern era, discovery of the role of miRNAs in the development of the cardiovascular system and pathogenesis of related diseases has reformed our perspective on appropriate treatment modalities. Nevertheless, despite the remarkable array of research on miRNAs in physiological and pathological state of the cardiovascular system, we have simply just begun to clarify the complexity of the heart and vascular system. The ever-growing body of research on the role that miRNAs play in diverse biological processes of the cardiovascular system will surely enable us to further scrutinize their functions and targets and to determine the miRNA-mRNA and miRNA-ncRNA complex interactions. 

The discovery of microRNA-based drugs is considered one of the most breathtaking therapeutic advances in biopharmaceuticals that will enter into commercial space as upcoming medicines. The optimization of some limitations like safety, efficacy, target selection, and delivery technologies, as well as clinical trial design and commercial considerations, will be in need of producing successful drugs. Furthermore, uncovering novel signaling mechanisms in the miRNA-based regulation will undoubtedly boost our knowledge about the management of the cardiovascular diseases and introducing novel candidate miRNAs for therapeutic approaches.

## References

[1] Fuster V, Kelly BB (2010). Promoting cardiovascular health in the developing world: a critical challenge to achieve global health.

[2] Hoffman JI, Kaplan S (2002). The incidence of congenital heart disease. JACC CardioOncol.

[3] Tennant PW, Pearce MS, Bythell M, Rankin J (2010). 20-year survival of children born with congenital anomalies: a population-based study. Lancet.

[4] Sun C, Kontaridis M (2018). Physiology of Cardiac Development: From Genetics to Signaling to Therapeutic Strategies. Curr Opin Physiol.

[5] Van Den Berg G, Moorman AF (2009). Concepts of cardiac development in retrospect. Pediatr Cardiol.

[6] Mandel EM, Callis TE, Wang D-Z, Conlon FL (2005). Transcriptional mechanisms of congenital heart disease. Drug Discov Today Dis Mech.

[7] Cecchetto A, Rampazzo A, Angelini A, Bianco LD, Padalino M, Stellin G (2010). From molecular mechanisms of cardiac development to genetic substrate of congenital heart diseases. Future Cardiol.

[8] Meyers EN, Martin GR (1999). Differences in left-right axis pathways in mouse and chick: functions of FGF8 and SHH. Science.

[9] Van Rooij E, Olson EN (2012). MicroRNA therapeutics for cardiovascular disease: opportunities and obstacles. Nat Rev Drug Discov.

[10] Albinsson S, Suarez Y, Skoura A, Offermanns S, Miano JM, Sessa WC (2010). MicroRNAs are necessary for vascular smooth muscle growth, differentiation, and function. Arterioscler Thromb Vasc Biol.

[11] Bartel DP, Chen C-Z (2004). Micromanagers of gene expression: the potentially widespread influence of metazoan microRNAs. Nat Rev Genet.

[12] Small EM, Olson EN (2011). Pervasive roles of microRNAs in cardiovascular biology. Nature.

[13] Lee RC, Feinbaum RL, Ambros V (1993). The C. elegans heterochronic gene lin-4 encodes small RNAs with antisense complementarity to lin-14. Cell.

[14] Wightman B, Ha I, Ruvkun G (1993). Posttranscriptional regulation of the heterochronic gene lin-14 by lin-4 mediates temporal pattern formation in C. elegans. Cell.

[15] Ha M, Kim VN (2014). Regulation of microRNA biogenesis. Nat Rev Mol Cell Biol.

[16] Lee Y, Kim M, Han J, Yeom KH, Lee S, Baek SH (2004). MicroRNA genes are transcribed by RNA polymerase II. EMBO J.

[17] Rodriguez A, Griffiths-Jones S, Ashurst JL, Bradley A (2004). Identification of mammalian microRNA host genes and transcription units. Genome Res.

[18] Conrad T, Marsico A, Gehre M, Ørom UA (2015). Microprocessor activity controls differential miRNA biogenesis in vivo. Cell Rep.

[19] Murchison EP, Hannon GJ (2004). miRNAs on the move: miRNA biogenesis and the RNAi machinery. Curr Opin Cell Biol.

[20] Kim VN, Han J, Siomi MC (2009). Biogenesis of small RNAs in animals. Nat Rev Mol Cell Biol.

[21] Hutvágner G, Zamore PD (2002). A microRNA in a multiple-turnover RNAi enzyme complex. Science.

[22] Chen J-F, Murchison EP, Tang R, Callis TE, Tatsuguchi M, Deng Z (2008). Targeted deletion of Dicer in the heart leads to dilated cardiomyopathy and heart failure. Proc Natl Acad Sci U S A.

[23] Zhao Y, Ransom JF, Li A, Vedantham V, von Drehle M, Muth AN (2007). Dysregulation of cardiogenesis, cardiac conduction, and cell cycle in mice lacking miRNA-1-2. Cell.

[24] da Costa Martins PA, Bourajjaj M, Gladka M, Kortland M, van Oort RJ, Pinto YM (2008). Conditional dicer gene deletion in the postnatal myocardium provokes spontaneous cardiac remodeling. Circulation.

[25] Cordes KR, Srivastava D (2009). MicroRNA regulation of cardiovascular development. Circ Res.

[26] Li B, Meng X, Zhang L (2018). MicroRNAs and cardiac stem cells in heart development and disease. Drug Discov Today.

[27] Vacante F, Denby L, Sluimer JC, Baker AH (2018). The function of miR-143, miR-145 and the miR-143 host gene in cardiovascular development and disease. Vascul Pharmacol.

[28] Heidersbach A, Saxby C, Carver-Moore K, Huang Y, Ang Y-S, de Jong PJ (2013). microRNA-1 regulates sarcomere formation and suppresses smooth muscle gene expression in the mammalian heart. Elife.

[29] Eulalio A, Mano M, Dal Ferro M, Zentilin L, Sinagra G, Zacchigna S (2012). Functional screening identifies miRNAs inducing cardiac regeneration. Nature.

[30] Wei Y, Peng S, Wu M, Sachidanandam R, Tu Z, Zhang S (2014). Multifaceted roles of miR-1s in repressing the fetal gene program in the heart. Cell Res.

[31] Chen J, Huang Z-P, Seok HY, Ding J, Kataoka M, Zhang Z (2013). mir-17–92 cluster is required for and sufficient to induce cardiomyocyte proliferation in postnatal and adult hearts. Circ Res.

[32] Van Rooij E, Quiat D, Johnson BA, Sutherland LB, Qi X, Richardson JA (2009). A family of microRNAs encoded by myosin genes governs myosin expression and muscle performance. Dev Cell.

[33] Rao PK, Kumar RM, Farkhondeh M, Baskerville S, Lodish HF (2006). Myogenic factors that regulate expression of muscle-specific microRNAs. Proc Natl Acad Sci U S A.

[34] Chen J-F, Mandel EM, Thomson JM, Wu Q, Callis TE, Hammond SM (2006). The role of microRNA-1 and microRNA-133 in skeletal muscle proliferation and differentiation. Nat Genet.

[35] Zhao Y, Samal E, Srivastava D (2005). Serum response factor regulates a muscle-specific microRNA that targets Hand2 during cardiogenesis. Nature.

[36] Zhao Y, Jaber V, Percy ME, Lukiw WJ (2017). A microRNA cluster (let-7c miRNA-99a, miRNA-125b miRNA-155 and miRNA-802) encoded at chr21q21 1-chr21q21 3 and the phenotypic diversity of Down’s syndrome (DS; trisomy 21). J Nat Sci.

[37] Ivey KN, Muth A, Arnold J, King FW, Yeh R-F, Fish JE (2008). MicroRNA regulation of cell lineages in mouse and human embryonic stem cells. Cell stem cell.

[38] Weiss A, Leinwand LA (1996). The mammalian myosin heavy chain gene family. Annu Rev Cell Dev Biol.

[39] Callis TE, Pandya K, Seok HY, Tang R-H, Tatsuguchi M, Huang ZP (2009). MicroRNA-208a is a regulator of cardiac hypertrophy and conduction in mice. J Clin Invest.

[40] Van Rooij E, Sutherland LB, Qi X, Richardson JA, Hill J, Olson EN (2007). Control of stress-dependent cardiac growth and gene expression by a microRNA. Science.

[41] Baldwin KM, Haddad F (2001). Invited review: effects of different activity and inactivity paradigms on myosin heavy chain gene expression in striated muscle. J Appl Physiol.

[42] Porrello ER (2013). microRNAs in cardiac development and regeneration. Clin Sci (Lond).

[43] Wang J, Greene SB, Bonilla-Claudio M, Tao Y, Zhang J, Bai Y (2010). Bmp signaling regulates myocardial differentiation from cardiac progenitors through a MicroRNA-mediated mechanism. Dev Cell.

[44] Chen J, Huang Z-P, Seok H, Ding J, Kataoka M, Zhang Z (2013). mir-17-92 cluster is required for and sufficient to induce cardiomyocyte proliferation in postnatal and adult hearts. Circ Res.

[45] Ai F, Zhang Y, Peng B (2016). miR-20a regulates proliferation, differentiation and apoptosis in P19 cell model of cardiac differentiation by targeting Smoothened. Biol Open.

[46] Xiang R, Lei H, Chen M, Li Q, Sun H, Ai J (2012). The miR-17-92 cluster regulates FOG-2 expression and inhibits proliferation of mouse embryonic cardiomyocytes. Braz J Med Biol Res.

[47] Tevosian SG, Deconinck AE, Tanaka M, Schinke M, Litovsky SH, Izumo S (2000). FOG-2, a cofactor for GATA transcription factors, is essential for heart morphogenesis and development of coronary vessels from epicardium. Cell.

[48] Porrello ER, Johnson BA, Aurora AB, Simpson E, Nam Y-J, Matkovich SJ (2011). MiR-15 family regulates postnatal mitotic arrest of cardiomyocytes. Circ Res.

[49] Kolpa HJ, Peal DS, Lynch SN, Giokas AC, Ghatak S, Misra S (2013). miR-21 represses Pdcd4 during cardiac valvulogenesis. Development.

[50] Thum T, Galuppo P, Wolf C, Fiedler J, Kneitz S, van Laake LW (2007). MicroRNAs in the human heart. Circulation.

[51] Maleki M, Alizadehasl A, Haghjoo M (2017). Practical cardiology.

[52] Khairy P, Ionescu-Ittu R, Mackie AS, Abrahamowicz M, Pilote L, Marelli AJ (2010). Changing mortality in congenital heart disease. J Am Coll Cardiol.

[53] Bensemlali M, Bajolle F, Ladouceur M, Fermont L, Lévy M, Le Bidois J (2016). Associated genetic syndromes and extracardiac malformations strongly influence outcomes of fetuses with congenital heart diseases. Arch Cardiovasc Dis.

[54] Li J, Cao Y, Ma X-j, Wang H-j, Zhang J, Luo X (2013). Roles of miR-1-1 and miR-181c in ventricular septal defects. Int J Cardiol.

[55] Das S, Bedja D, Campbell N, Dunkerly B, Chenna V, Maitra A (2014). miR-181c regulates the mitochondrial genome, bioenergetics, and propensity for heart failure in vivo. PLoS one.

[56] Smith T, Rajakaruna C, Caputo M, Emanueli C (2015). MicroRNAs in congenital heart disease. Ann Transl Med.

[57] Li D, Ji L, Liu L, Liu Y, Hou H, Yu K (2014). Characterization of circulating microRNA expression in patients with a ventricular septal defect. PLoS One.

[58] Li J, Dong X, Wang Z, Wu J (2014). MicroRNA-1 in cardiac diseases and cancers. Korean J Physiol Pharmacol.

[59] O’Brien JE Jr, Kibiryeva N, Zhou XG, Marshall JA, Lofland GK, Artman M (2012). Noncoding RNA expression in myocardium from infants with tetralogy of Fallot. Circ Cardiovasc Genet.

[60] Bittel DC, Kibiryeva N, Marshall JA, O’Brien JE (2014). MicroRNA-421 dysregulation is associated with tetralogy of Fallot. Cells.

[61] Liu N, Williams AH, Kim Y, McAnally J, Bezprozvannaya S, Sutherland LB (2007). An intragenic MEF2-dependent enhancer directs muscle-specific expression of microRNAs 1 and 133. Proc Natl Acad Sci U S A.

[62] Liu N, Bezprozvannaya S, Williams AH, Qi X, Richardson JA, Bassel-Duby R (2008). microRNA-133a regulates cardiomyocyte proliferation and suppresses smooth muscle gene expression in the heart. Genes Dev.

[63] Ventura A, Young AG, Winslow MM, Lintault L, Meissner A, Erkeland SJ (2008). Targeted deletion reveals essential and overlapping functions of the miR-17∼ 92 family of miRNA clusters. Cell.

[64] Kuhn DE, Nuovo GJ, Martin MM, Malana GE, Pleister AP, Jiang J (2008). RETRACTED: Human chromosome 21-derived miRNAs are overexpressed in down syndrome brains and hearts. Biochem Biophys Res Commun.

[65] Latronico MV, Condorelli G (2009). MicroRNAs and cardiac pathology. Nat Rev Cardiol.

[66] Han J, Lee Y, Yeom K-H, Kim Y-K, Jin H, Kim VN (2004). The Drosha-DGCR8 complex in primary microRNA processing. Genes Dev.

[67] Omran A, Elimam D, Webster KA, Shehadeh LA, Yin F (2013). MicroRNAs: a new piece in the paediatric cardiovascular disease puzzle. Cardiol Young.

[68] Kim T-K, Hemberg M, Gray JM Enhancer RNAs: a class of long noncoding RNAs synthesized at enhancers. Cold Spring Harb Perspect Biol.

[69] Zheng D, Xu L, Sun L, Feng Q, Wang Z, Shao G (2014). Comparison of the ventricle muscle proteome between patients with rheumatic heart disease and controls with mitral valve prolapse: HSP 60 may be a specific protein in RHD. Biomed Res Int.

[70] Guilherme L, Kalil J (2013). Rheumatic heart disease: molecules involved in valve tissue inflammation leading to the autoimmune process and anti-s pyogenes vaccine. Front Immunol.

[71] Peterlongo P, Caleca L, Cattaneo E, Ravagnani F, Bianchi T, Galastri L (2011). The rs12975333 variant in the miR-125a and breast cancer risk in Germany, Italy, Australia and Spain. J Med Genet.

[72] Li N, Lian J, Zhao S, Zheng D, Yang X, Huang X (2015). Detection of differentially expressed micrornas in rheumatic heart disease: miR-1183 and miR-1299 as potential diagnostic biomarkers. Biomed Res Int.

[73] Hu M, Wei X, Li M, Tao L, Wei L, Zhang M (2019). Circular RNA expression profiles of persistent atrial fibrillation in patients with rheumatic heart disease. Anadolu Kardiyol Derg.

[74] Paul P, Chakraborty A, Sarkar D, Langthasa M, Rahman M, Bari M (2018). Interplay between miRNAs and human diseases. J Cell Physiol.

[75] Gumus G, Giray D, Bobusoglu O, Tamer L, Karpuz D, Hallioglu O (2018). MicroRNA values in children with rheumatic carditis: a preliminary study. Rheumatol Int.

[76] Zhu QY, Liu Q, Chen JX, Lan K, Ge BX (2010). MicroRNA-101 targets MAPK phosphatase-1 to regulate the activation of MAPKs in macrophages. J Immunol.

[77] Lu Q, Sun Y, Duan Y, Li B, Xia J, Yu S (2018). Comprehensive microRNA profiling reveals potential augmentation of the IL1 pathway in rheumatic heart valve disease. BMC Cardiovasc Disord.

[78] Dong H, Sun Y, Shan F, Sun Q, Yang B (2015). Down-regulation of miR-101 contributes to rheumatic heart disease through up-regulating TLR2. Med Sci Monit.

[79] Huang Y, Li J (2015). MicroRNA208 family in cardiovascular diseases: therapeutic implication and potential biomarker. J Physiol Biochem.

[80] Knabel MK, Ramachandran K, Karhadkar S, Hwang HW, Creamer TJ, Chivukula RR (2015). Systemic delivery of scAAV8-encoded MiR-29a ameliorates hepatic fibrosis in carbon tetrachloride-treated mice. PLoS One.

[81] Lovric J, Mano M, Zentilin L, Eulalio A, Zacchigna S, Giacca M (2012). Terminal differentiation of cardiac and skeletal myocytes induces permissivity to AAV transduction by relieving inhibition imposed by DNA damage response proteins. Mol Ther.

[82] Thum T (2012). MicroRNA therapeutics in cardiovascular medicine. EMBO Mol Med.

[83] Canfran-Duque A, Rotllan N, Zhang X, Fernandez-Fuertes M, Ramirez-Hidalgo C, Araldi E (2017). Macrophage deficiency of miR-21 promotes apoptosis, plaque necrosis, and vascular inflammation during atherogenesis. EMBO Mol Med.

[84] Malakootian M, Mirzadeh Azad F, Fouani Y, Taheri Bajgan E, Saberi H, Mowla SJ (2018). Anti-differentiation non-coding RNA, ANCR, is differentially expressed in different types of brain tumors. J Neurooncol.

[85] Jeng MY, Mumbach MR, Granja JM, Satpathy AT, Chang HY, Chang ALS (2019). Enhancer connectome nominates target genes of inherited risk variants from inflammatory skin disorders. J Invest Dermatol.

[86] Pivarcsi A, Széll M, Kemény L, Dobozy A, Bata-Csörgő Z (2001). Serum factors regulate the expression of the proliferation-related genes α5 integrin and keratin 1, but not keratin 10, in HaCaT keratinocytes. Arch Dermatol Res.

[87] Chen Y, Gao D-Y, Huang L (2015). In vivo delivery of miRNAs for cancer therapy: challenges and strategies. Adv Drug Deliv Rev.

[88] Olson EN (2014). MicroRNAs as therapeutic targets and biomarkers of cardiovascular disease. Sci Transl Med..

[89] Chakraborty C, Sharma AR, Sharma G, Doss CGP, Lee SS (2017). Therapeutic miRNA and siRNA: moving from bench to clinic as next generation medicine. Mol Ther Nucleic Acids.

